# Supplementary material for: Splice-Junction-Based Mapping of Alternative Isoforms in the Human Proteome
Source: Cell Rep. Author manuscript; Available in PMC 2020 Jan 15. (PMC6961840; doi:10.1016/j.celrep.2019.11.026)
Supplement: 2 [file NIHMS1546469-supplement-2.zip › DF1/PXD006675/InferiorVenaCava/InferiorVenaCava_1_CLTCL1_LQEHFQLQNLGINPANIGFSTLTMESDK.pdf]

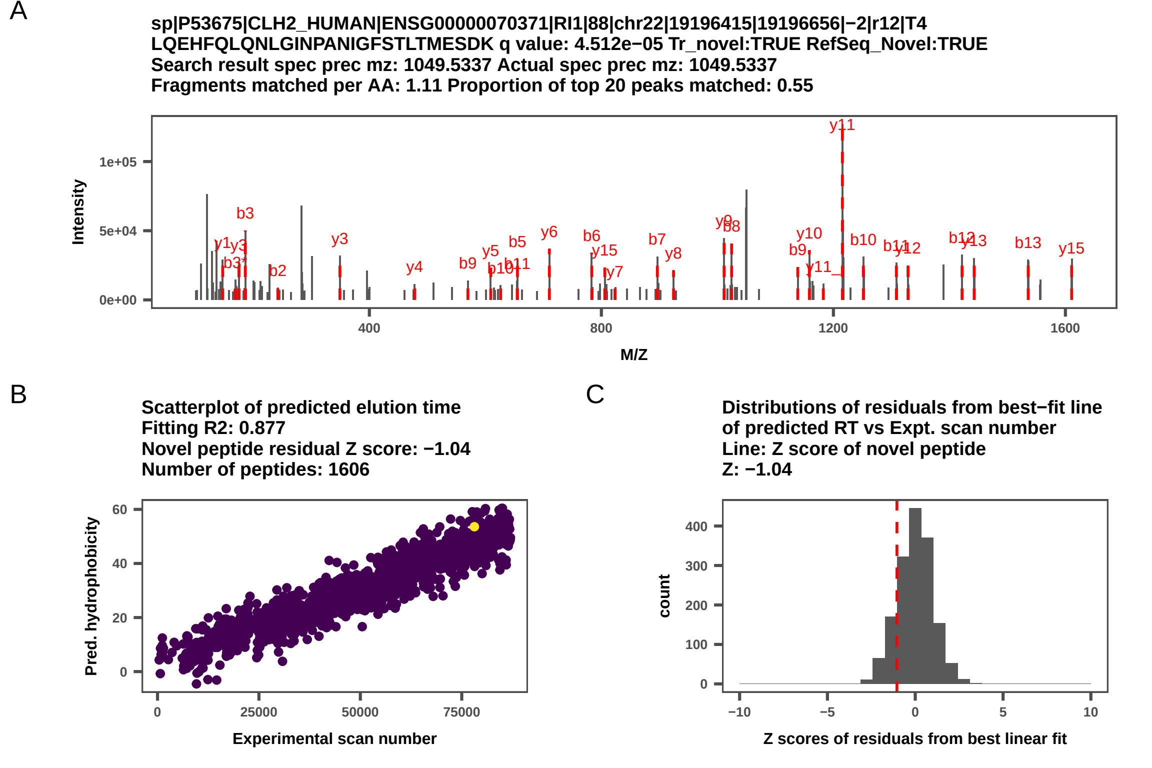

A
sp|P53675|CLH2_HUMAN|ENSG00000070371|RI1|88|chr22|19196415|19196656|−2|r12|T4
LQEHFQLQNLGINPANIGFSTLTMESDK q value: 4.512e−05 Tr_novel:TRUE RefSeq_Novel:TRUE
Search result spec prec mz: 1049.5337 Actual spec prec mz: 1049.5337
Fragments matched per AA: 1.11 Proportion of top 20 peaks matched: 0.55
Intensity
1e+05
5e+04
0e+00
M/Z
400
800
1200
1600
y11
b3
y1
y3
b3*
b2
y3
y4
b9
y5
b10
b11
b5
y6
b6
y15
y7
b7
y8
y9
b8
b9
y10
y11_
b10
b11
y12
b12
y13
b13
y15
B
Scatterplot of predicted elution time
Fitting R2: 0.877
Novel peptide residual Z score: −1.04
Number of peptides: 1606
Pred. hydrophobicity
60
40
20
0
0
25000
50000
75000
Experimental scan number
C
Distributions of residuals from best−fit line
of predicted RT vs Expt. scan number
Line: Z score of novel peptide
Z: −1.04
count
400
300
200
100
0
−10
−5
0
5
10
Z scores of residuals from best linear fit
